# Early growth patterns and cardiometabolic function at the age of 5 in a multiethnic birth cohort: the ABCD study

**DOI:** 10.1186/1471-2431-9-23

**Published:** 2009-03-24

**Authors:** Marieke de Beer, Manon van Eijsden, Tanja GM Vrijkotte, Reinoud JBJ Gemke

**Affiliations:** 1 Department of Pediatrics, EMGO Institute, Institute for Cardiovascular Research VU, VU University Medical Centre, Amsterdam, the Netherlands; 2 Department of Epidemiology, Documentation and Health Promotion, Public Health Service, Amsterdam, the Netherlands; 3 Department of Social Medicine, Academic Medical Centre, University of Amsterdam, Amsterdam, the Netherlands

## Abstract

**Background:**

The relation between fetal growth retardation and cardiovascular and metabolic diseases in later life has been demonstrated in many studies. However, debate exists around the potential independent role of postnatal growth acceleration. Furthermore, it is unknown whether a potential effect of growth acceleration on cardiovascular and metabolic function is confined to certain timeframes.

The present study assesses the (predictive) role of prenatal and postnatal growth on 5 components of cardiovascular and metabolic function in children aged 5. The potential association of timing of postnatal growth acceleration with these outcomes will be explored.

**Methods and design:**

Prospective multiethnic community-based cohort study of 8266 pregnancies (Amsterdam Born Children and their Development, ABCD study). Up till now, anthropometry of 5104 children from the original cohort was followed during the first 5 years of life, with additional information about birth weight, pregnancy duration, and various potential confounding variables.

At age 5, various components of cardiovascular and metabolic function are being measured. Outcome variables are body size, body composition and fat distribution, insulin sensitivity, lipid profile, blood pressure and autonomic regulation of cardiovascular function.

**Discussion:**

This study will be one of the first population-based prospective cohort studies to address the association between measures of both prenatal and postnatal growth and various components of cardiovascular and metabolic function. Specific attention is paid to the timing of acceleration in growth and its potential association with the outcome variables. Importantly, the longitudinal design of this study gives us the opportunity to gain more insight into growth trajectories associated with adverse outcomes in later life. If identified as an independent risk factor, this provides further basis for the hypothesis that accelerated growth during the first years of life is a modifiable factor for the prevention of cardiovascular and metabolic disorders in later life. Moreover, identification of specific vulnerable periods during development may reveal suitable timeframes for early interventions.

## Background

Although it is clear that genetic factors contribute to the propensity towards obesity and associated conditions, the dramatic increase in prevalence in the last decades underlines the role of environmental determinants. Recent findings associated with the hypothesis of Developmental Origins of Health and Disease (DOHaD) have pointed out that cardiovascular and metabolic diseases may originate from periconceptional and perinatal environmental factors.[1,2] More specifically, a series of epidemiological studies in several countries has documented that subjects with fetal growth retardation due to intrauterine malnutrition have a higher risk of developing hypertension, obesity, diabetes and coronary heart disease in later life. [3-10] In addition, prematurity has recently been demonstrated as an independent risk factor for high blood pressure and cardiovascular diseases in later life.[11] Furthermore, evolving evidence suggests an independent role of rapid postnatal growth (also referred to as "catch-up growth") on cardiovascular and metabolic diseases in later life (the so called accelerated growth hypothesis). Rapid growth in postnatal life may be driven by the aim to compensate for antenatal growth retardation or prematurity which may subsequently increase the adverse effect on cardiovascular disease risk in later life.[3,5,7,12-14] However, the occurrence of accelerated postnatal growth is not limited to babies born growth retarded or premature, it also occurs in full term, normal birth weight infants, with comparable adverse associations with cardiovascular and metabolic function in later life.[15]

The exact timing of growth acceleration incurring associated cardiovascular and metabolic diseases has not been established unequivocally. It is plausible that the moment of occurrence is of greater importance than the magnitude of growth acceleration itself. [16-19] Studies addressing this subject are inconclusive. While Singhal et al. and Ong et al. reported an adverse effect of accelerated infant growth on cardiovascular and metabolic function, the Helsinki observations showed that the combination of slow growth in utero, low weight gain in infancy (thinness at 1 year) and growth acceleration in childhood is a strong predictor of coronary heart disease.[6,8,18,20,21]

The pathophysiological mechanisms linking early growth and cardiovascular and metabolic function in later life remain elusive. Impaired fetal growth as such may not be the cause of the long-term consequences, but rather constitute a marker of a coordinated fetal response to a restricted intrauterine environment. This coordinated fetal response may result in persistent alterations in physiologic and metabolic homeostatic set points, which in a rich extrauterine environment may be followed by accelerated postnatal growth and subsequent increased risk of cardiovascular and metabolic diseases in later life. Notably, fetal growth retardation may not always be adequately represented by a reduced birth weight.[1] Moreover, retarded fetal growth may be associated with alterations in organ development which in turn are associated with adult cardiovascular and metabolic diseases. This may be enhanced if retarded fetal growth is followed by accelerated postnatal growth. More specifically, associations have been found between fetal growth retardation and a reduction in the number of nephrons[22] (associated with hypertension), reduced abdominal circumference[23] (as an indication of liver size and cholesterol regulation), and a reduction in the number of pancreatic β cells[24] (leading to decreased production of insulin, and potentially to type 2 diabetes). Also, thinness at birth may be associated with abnormalities in muscle structure and function which may have long term consequences that interfere with insulin's ability to promote glucose uptake in skeletal muscle (leading to insulin resistance).[25]

If identified as an independent risk factor in community based studies, accelerated growth during the first years of life may be an important modifiable factor for the prevention of obesity, and other cardiovascular and metabolic diseases in later life.

### Hypotheses

#### Primary outcome variable

• Impaired intrauterine growth, with subsequent accelerated growth during the immediate postnatal period, infancy and childhood is an independent determinant of body size (BMI), body composition (fat percentage, fat-free mass) and fat distribution (waist circumference, waist-to-height-ratio) at the age of 5.

#### Secondary outcome measures

• Impaired intrauterine growth, with subsequent accelerated growth during the immediate postnatal period, infancy and childhood is an independent determinant of insulin sensitivity at the age of 5.

• Impaired intrauterine growth, with subsequent accelerated growth during the immediate postnatal period, infancy and childhood is an independent determinant of lipid profile at the age of 5.

• Impaired intrauterine growth, with subsequent accelerated growth during the immediate postnatal period, infancy and childhood is an independent determinant of blood pressure at the age of 5.

• Impaired intrauterine growth, with subsequent accelerated growth during the immediate postnatal period, infancy and childhood is an independent determinant of autonomic regulation of cardiovascular function at the age of 5.

### Aims

• To examine the role of fetal growth (retardation) and subsequent (accelerated) postnatal growth on 5 different components of cardiovascular and metabolic function at 5 years, incorporating potential effect-modifying variables like gender and prematurity and various potential confounding variables.

• To determine the time period of postnatal growth acceleration which is most associated with these components of cardiovascular and metabolic function.

## Methods and design

### Procedure (figure 1)

**Figure 1:**
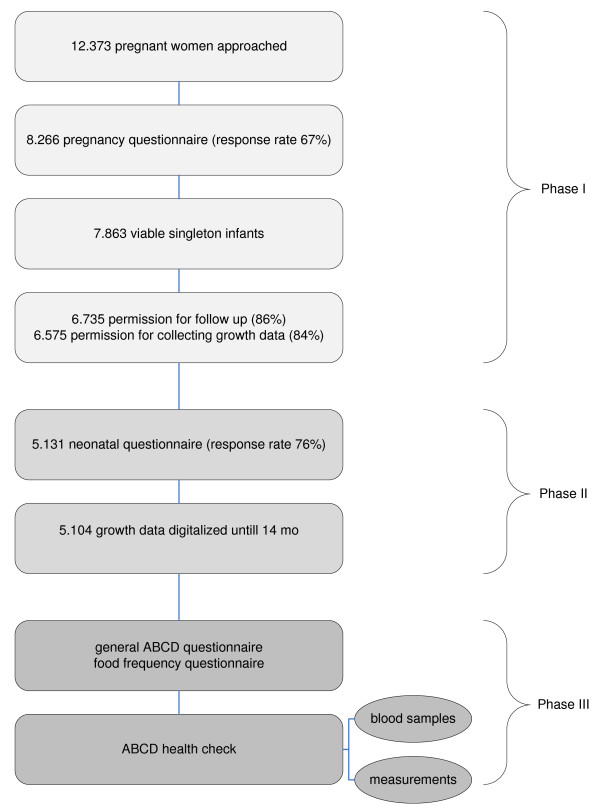
**Procedure**.

#### Phase I and II

The ABCD study is a prospective cohort study, a collaborative effort of the Municipal Health Service (GGD), all hospitals and midwife practices in Amsterdam and a research group hosted at the GGD/Amsterdam Medical Centre (AMC)/VU university medical centre Amsterdam (VUmc). The study focuses on the explanatory role of lifestyle and dietary habits during pregnancy in birth outcomes and future health of the child, with specific attention to ethnicity .

Between January 2003 and March 2004, all pregnant women living in Amsterdam were invited to participate in this study at their first visit to an obstetric caregiver. A questionnaire, covering sociodemographic data, obstetric history, lifestyle, dietary habits, and psychosocial factors, was sent to the pregnant woman's home address, this was 2 weeks after their fist visit (12–14^th ^week of pregnancy). Questionnaires were returned by 8.266 women (response rate: 67%). From this group 7.863 gave birth to a viable singleton infant.

Pregnancy duration, gender and birth weight were obtained from Youth Health Care centres which perform neonatal screening on congenital inborn errors of metabolism in all Dutch newborns. A standardised birth weight was calculated using the most recent Dutch reference values obtained from the Dutch perinatal registration . Birth weight was standardised for gender, pregnancy duration and parity. Birth weight SDS is interpreted as an expression of intrauterine growth.

Three months after delivery another questionnaire was sent to the mothers who had given permission to follow the health status of the child for further research (n = 6.735) with questions concerning the course of pregnancy and delivery, questions about health, development and growth of the baby, and questions about lifestyle of the mother during and after pregnancy. With 5.131 women returning the questionnaire the response rate was 76%.

Until the age of 14 months length and weight of these children were routinely collected at the Youth Health Care registration of the Public Health Service in Amsterdam. These measurements took place during on average 9 regular follow-up moments and were performed by well trained nurses.

6.575 mothers gave permission to follow up the growth data of their child. Up till now, growth data of 5.104 of these children are digitized.

#### Phase III

Phase three of the study has started in the summer of 2008. Around two weeks after their ABCD-child's 5^th ^birthday, mothers who had initially given permission for follow up are sent a questionnaire in which they are also asked for permission on participation of their child in the ABCD health check.

Two weeks before the health check mothers receive a notifying letter and an additional self administered food frequency questionnaire (FFQ).

The health check is situated at the children's primary school (which are located all over Amsterdam). One half hour before the school begins a blood sample is taken from the children by finger prick. The various measurements take place during the rest of the day.

### Outcome variables and potential confounding and effect modifying variables (table 1)

**Table 1:** Outcome variables and potential confounding/effect modifying variables

**Cardiometabolic risk profile**	**Direct outcome** **variables**	**Derived outcome variables**	**Potential confounding/effect modifying (EM) variables**
Body size, body composition and fat distribution	WC	BMI *(primary outcome variable)*	gender (EM)
		WHtR	prematurity (EM)
		Fat%	Family history positive for CVD or metabolic diseases
		FFM	maternal smoking
			maternal and paternal BMI
Insulin sensitivity	Gluc	HOMA-IR	maternal educational level and cohabitant status
		HOMA-%B	physical exercise
			sedentary behaviour
Lipid profile	TC		amount of sleep
	HDL		caloric intake
	TG		eating behaviour
			maternal attitude towards child weight
Blood pressure	SP	MAP	
	DP		
Autonomic regulation of cardiovascular function		HR	
		RMSSD	
		PEP	

BMI = body mass index; CVD = cardiovascular disease; DP = diastolic blood pressure; Fat% = fat percentage; FFM = fat-free mass; Gluc = fasting plasma glucose; HDL = high density lipoprotein cholesterol; HOMA-IR = homeostasis model assessment-insulin resistance; HOMA-%B = homeostasis model assessment β cell function; HR = heart rate; MAP = mean arterial pressure; PEP = pre ejection period; RMSSD = root mean square of successive inter beat intervals; SP = systolic blood pressure; TC = total cholesterol; TG = triglycerides; WC = waist circumference; WHtR = waist-height-ratio

#### Questionnaires

The general ABCD-questionnaire contains items about the child's health, development and behaviour (i.e. physical exercise, sedentary behaviour, amount of sleep, and the Child Feeding Questionnaire (CFQ).[26] Furthermore, it contains items regarding mother's attitude towards the child's weight, maternal lifestyle and parents' and grandparents' history of cardiovascular (CVD) and metabolic disorders.

The FFQ is developed by TNO Food (Zeist, the Netherlands). It consists of 77 food items, for which the frequency of consumption and portion size are to be estimated. Results of a validation study comparing the TNO-FFQ with the gold standard doubly labelled water are promising; the Pearson correlation coefficient between energy intake (TNO-FFQ) and energy expenditure was 0.62 (to be published).

#### Blood sampling and processing

Capillary blood is collected in a well validated collection kit which is developed for ambulatory purposes (Demecal, Haarlem, The Netherlands).[27] Fasting plasma glucose (Gluc), C-peptide, total cholesterol (TC), high density lipoprotein cholesterol (HDL) and triglycerides (TG) are determined.

Outcome measures are Gluc, insulin resistance and β-cell function (HOMA-IR, HOMA-%B)[28], TC, HDL and TG (mmol/dl).

#### Anthropometrics

Height (Ht) is measured to the nearest millimetre using a Leicester portable height measure (Seca), and weight (Wt) to the nearest 100 gram using a Marsden weighing scale, model MS-4102. Waist circumference (WC) is measured midway between the costal border and the iliac crest to the nearest millimetre using a Seca measuring tape.

Outcome measures are WC, expressed in cm, waist-to-height ratio (WHtR) and body mass index (BMI). BMI is calculated as the individual's weight in kilograms divided by their height in meters squared.

#### Bioelectrical impedance analysis

Arm-to-leg bioelectrical impedance analysis (BIA) is measured twice using the Bodystat 1500 MDD machine (Bodystat Inc, Douglas, UK). Body fat percentage (Fat%) is calculated using the following equations, adapted from Kushner et al.[29] and Lohman.[30]:

(1) TBW = 0,59 Ht^2^/R_50 _+ 0,065 Wt +0,04

(2) FFM = *c *TBW

(3) Fat % = ((Wt - FFM)/Wt) * 100%

In which TBW is total body water, R_50 _is resistance at 50 kHz, FFM is fat free mass and c is 1,30 (100/77) for males and 1,28 (100/78) for females.

Outcome measures are Fat% and FFM.

#### Blood pressure measurement

Blood pressure is measured 4 times on the right arm: 2 times in a supine position after 5 minutes of rest and 2 times in a sitting position after 5 minutes of rest. The device used is the Omron 705 IT (Omron Healthcare Inc, Bannockburn, IL, USA) with its small cuff (arm circumference 17–22 cm).

Outcome measures are systolic pressure (SP), diastolic pressure (DP), and mean arterial pressure (MAP), all expressed in mmHg.

MAP is calculated using the following equation:

MAP = DP + 1/3 (SP-DP)

#### Measurement of autonomic regulation of cardiovascular function

Heart rate (HR), root mean square of successive inter beat intervals (RMSSD) and pre ejection period (PEP) are different measures of cardiac autonomic control which can be obtained non-invasively and in an ambulatory setting with various devices. We used the VU University Ambulatory Monitoring System (VU-AMS) to assess the various measures, in a supine position during 7 minutes after 1 minute of rest and in a sitting position during 7 minutes after 1 minute of rest. The VU-AMS continuously records an electrocardiogram (ECG) and an impedance cardiogram (ICG).

Reliability and validity aspects and recording methodology of the VU-AMS have been described previously.[31] A continuous time series of R wave to R wave intervals is derived on line from a three lead ECG. HR can be directly derived from the time between two adjacent R waves. Vagal tone is assessed by the root mean square of successive differences in these interbeat intervals (RMSSD), that has previously been proven to be a valid index of vagal tone. (task force, 1996). The ICG is obtained by 4 electrodes. The B-point signals the opening of the aortic valves and is scored in the ICG signal. PEP is the time interval between the onset of ventricular depolarisation (the Q wave onset in the ECG) and the opening of the aortic valves (B-point in ICG) and is considered to be an adequate surrogate measure for sympathic nervous system activity.[31]

### Data handling and statistical analysis

#### Data analysis for 5 outcomes of cardiovascular and metabolic function

Similar to other studies analysing growth trajectories, follow-up in growth (weight and height) is divided in 3 time periods for each individual: immediate growth (between 1 and 6 months), infant growth (between 6 months and 14 months), and childhood growth (between 14 months and 5 years) [17]. For each time period the average standardised growth velocity is calculated by subtracting the standardized weight or height at the earlier moment in time from the standardized weight or height at the later moment in time, resulting in a ΔSDS. Dutch reference standards are used in expressing weight and height as standard deviation scores (SDS).[32] Growth acceleration in weight and height, respectively, are both defined as ΔSDS>0.67. SDS 0.67 represents the width of each percentile band on standard growth charts (that is P2–P9, P9–P25, P25–P50, etc), and consequently this is the most commonly used indicator of clinically significant accelerated growth.[14,33]

Analysis of the 5 years outcome measures are undertaken using linear and/or logistic regression with the 4 random effects (standardised birth weight, and ΔSDS (standardised growth velocity) for each of the 3 time periods) as exposures (expressed as continuous variables). We will examine the effects of fetal and postnatal growth (both weight and height) with 3 models to account for potential confounders: (1) mutual adjustment for all of the growth measures; (2) as in model 1 but with the addition of confounding variables, to be determined by univariate analysis; (3) as in model 2 but with the addition of potential effect modifiers (gender and prematurity), to be determined by assessment of interaction.

All aforementioned analyses are repeated with the exposure variables dichotomised; standardised birth weight is divided in > and ≤ average and standardised growth velocity (ΔSDS) in > and ≤ 0.67.

#### Power analysis

For power calculations BMI is chosen as the primary outcome variable. Based on 90% power (1-β), to detect a 0.5 point difference in BMI between children with and without growth acceleration in weight (α = 0.05, two-sided), a total of 850 children are required. In the calculation we have accounted for the prevalence of accelerated growth in weight in the first 6 months, which in our population is around 19%. In comparison, Karaolis-Danckert et al. found a prevalence of growth acceleration of around 29% in the first two years of life.[15]

### Inclusion/exclusion criteria

Multiple births were excluded from the analysis.

### Ethics

Approval of the study was obtained from the Central Committee on Research involving Human Subjects in the Netherlands, the Medical Ethical Committees of participating hospitals, and from the Registration Committee of the Municipality of Amsterdam.

## Discussion

Emerging evidence indicates that adult degenerative diseases are related to different patterns of fetal, infant, and childhood growth. Retarded growth during fetal life and accelerated weight gain during infancy and childhood appear to be associated with the development of cardiovascular disease and type 2 diabetes in adult life. Unfortunately it is not clear what optimal growth is and how it can be achieved. Although deviations from normal early growth patterns become regarded as important risk factors for adult disease, they are not causative factors as such. As early life risk factors, they are to a large degree modified by various variables during childhood and adult life.

Our study is one of the first large population based prospective cohort studies which will address the association of prenatal growth retardation and postnatal growth acceleration with outcome variables including various components of cardiovascular and metabolic function. Specific attention is paid to the exact timing of growth acceleration and its association with these outcome variables. Importantly, the longitudinal design of this study offers us the opportunity to gain more insight into growth trajectories associated with adverse outcomes in later life.

Several well-established components of (albeit adult) cardiovascular and metabolic function will be addressed in our study, including obesity, insulin sensitivity, lipid profile and blood pressure. As obesity in children is not easy to determine and cut-off values vary with age, we will address body composition (fat mass and fat-free mass) and fat distribution (waist circumference and waist-height-ratio) in addition to BMI. Furthermore, the association of autonomic regulation of cardiovascular function with different patterns of growth in early life will be assessed. To our knowledge, this is innovative in the field of cardiovascular research. If an association is established, this suggests that changes in autonomic regulation may be important in linking early life factors and cardiovascular diseases in later life.

If identified as an important independent risk factor in this large community based study, this provides further ground for the hypothesis that accelerated growth during the first years of life comprise an important modifiable factor for the prevention of obesity, and other cardiovascular and metabolic diseases in later life. Moreover, identification of vulnerable periods during development may reveal suitable timeframes for early interventions.

## Competing interests

The authors declare that they have no competing interests.

## Authors' contributions

RG obtained funding. All authors are responsible for the concept and design of the study. MB drafted the manuscript, RG, ME, and TV revisited the draft versions.

## Pre-publication history

The pre-publication history for this paper can be accessed here:



## References

[B1] Gluckman PD, Hanson MA, Cooper C, Thornburg KL (2008). Effect of in utero and early-life conditions on adult health and disease. N Engl J Med.

[B2] Fernandez-Twinn DS, Ozanne SE (2006). Mechanisms by which poor early growth programs type-2 diabetes, obesity and the metabolic syndrome. Physiol Behav.

[B3] Huxley RR, Shiell AW, Law CM (2000). The role of size at birth and postnatal catch-up growth in determining systolic blood pressure: a systematic review of the literature. J Hypertens.

[B4] Mi J, Law C, Zhang KL, Osmond C, Stein C, Barker D (2000). Effects of infant birthweight and maternal body mass index in pregnancy on components of the insulin resistance syndrome in China. Ann Intern Med.

[B5] Eriksson JG, Forsen T, Tuomilehto J, Osmond C, Barker D (2000). Fetal and childhood growth and hypertension in adult life. Hypertension.

[B6] Eriksson JG, Forsen T, Tuomilehto J, Osmond C, Barker DJ (2001). Early growth and coronary heart disease in later life: longitudinal study. BMJ.

[B7] Eriksson JG, Forsen T, Tuomilehto J, Jaddoe VW, Osmond C, Barker DJ (2002). Effects of size at birth and childhood growth on the insulin resistance syndrome in elderly individuals. Diabetologia.

[B8] Ong KK, Dunger DB (2004). Birth weight, infant growth and insulin resistance. Eur J Endocrinol.

[B9] Forsen T, Eriksson J, Tuomilehto J, Reunanen A, Osmond C, Barker D (2000). The fetal and childhood growth of persons who develop type 2 diabetes. Ann Intern Med.

[B10] Ong KK, Petry CJ, Emmett PM, Sandhu MS, Kiess W, Hales CN (2004). Insulin sensitivity and secretion in normal children related to size at birth, postnatal growth, and plasma insulin-like growth factor-I levels. Diabetologia.

[B11] Leon DA, Johansson M, Rasmussen F (2000). Gestational age and growth rate of fetal mass are inversely associated with systolic blood pressure in young adults: an epidemiologic study of 165,136 Swedish men aged 18 years. Am J Epidemiol.

[B12] Ong KK, Preece MA, Emmett PM, Ahmed ML, Dunger DB (2002). Size at birth and early childhood growth in relation to maternal smoking, parity and infant breast-feeding: longitudinal birth cohort study and analysis. Pediatr Res.

[B13] Eriksson JG, Forsen T, Tuomilehto J, Winter PD, Osmond C, Barker DJ (1999). Catch-up growth in childhood and death from coronary heart disease: longitudinal study. BMJ.

[B14] Ong KK, Ahmed ML, Emmett PM, Preece MA, Dunger DB (2000). Association between postnatal catch-up growth and obesity in childhood: prospective cohort study. BMJ.

[B15] Karaolis-Danckert N, Buyken AE, Bolzenius K, Perim de FC, Lentze MJ, Kroke A (2006). Rapid growth among term children whose birth weight was appropriate for gestational age has a longer lasting effect on body fat percentage than on body mass index. Am J Clin Nutr.

[B16] Eriksson J (2001). Commentary: Early 'catch-up' growth is good for later health. Int J Epidemiol.

[B17] Ben-Shlomo Y, McCarthy A, Hughes R, Tilling K, Davies D, Smith GD (2008). Immediate postnatal growth is associated with blood pressure in young adulthood: the Barry Caerphilly Growth Study. Hypertension.

[B18] Singhal A, Cole TJ, Fewtrell M, Deanfield J, Lucas A (2004). Is slower early growth beneficial for long-term cardiovascular health?. Circulation.

[B19] Stettler N, Kumanyika SK, Katz SH, Zemel BS, Stallings VA (2003). Rapid weight gain during infancy and obesity in young adulthood in a cohort of African Americans. Am J Clin Nutr.

[B20] Singhal A, Lucas A (2004). Early origins of cardiovascular disease: is there a unifying hypothesis?. Lancet.

[B21] Eriksson JG, Forsen T, Tuomilehto J, Osmond C, Barker DJ (2003). Early adiposity rebound in childhood and risk of Type 2 diabetes in adult life. Diabetologia.

[B22] Mackenzie HS, Brenner BM (1995). Fewer nephrons at birth: a missing link in the etiology of essential hypertension?. Am J Kidney Dis.

[B23] Barker DJ, Martyn CN, Osmond C, Hales CN, Fall CH (1993). Growth in utero and serum cholesterol concentrations in adult life. BMJ.

[B24] Hales CN, Barker DJ (1992). Type 2 (non-insulin-dependent) diabetes mellitus: the thrifty phenotype hypothesis. Diabetologia.

[B25] Taylor DJ, Thompson CH, Kemp GJ, Barnes PR, Sanderson AL, Radda GK (1995). A relationship between impaired fetal growth and reduced muscle glycolysis revealed by 31P magnetic resonance spectroscopy. Diabetologia.

[B26] Birch LL, Fisher JO, Grimm-Thomas K, Markey CN, Sawyer R, Johnson SL (2001). Confirmatory factor analysis of the Child Feeding Questionnaire: a measure of parental attitudes, beliefs and practices about child feeding and obesity proneness. Appetite.

[B27] Gootjes J, Tel RM, Bergkamp FJ, Gorgels JP (2009). Laboratory evaluation of a novel capillary blood sampling device for measuring eight clinical chemistry parameters and HbA1c. Clin Chim Acta.

[B28] Wallace TM, Levy JC, Matthews DR (2004). Use and abuse of HOMA modeling. Diabetes Care.

[B29] Kushner RF, Schoeller DA, Fjeld CR, Danford L (1992). Is the impedance index (ht2/R) significant in predicting total body water?. Am J Clin Nutr.

[B30] Lohman TG (1989). Assessment of body composition in children. Pediatric Exercise Science.

[B31] Vrijkotte TG, van Doornen LJ, de Geus EJ (2004). Overcommitment to work is associated with changes in cardiac sympathetic regulation. Psychosom Med.

[B32] Fredriks AM, van Buuren S, Burgmeijer RJ, Meulmeester JF, Beuker RJ, Brugman E (2000). Continuing positive secular growth change in The Netherlands 1955–1997. Pediatr Res.

[B33] Ong KK, Loos RJ (2006). Rapid infancy weight gain and subsequent obesity: systematic reviews and hopeful suggestions. Acta Paediatr.

